# Association between benzodiazepine anxiolytic polypharmacy and concomitant psychotropic medications in Japan: a retrospective cross-sectional study

**DOI:** 10.3389/fpsyt.2024.1405049

**Published:** 2024-07-04

**Authors:** Masahiro Takeshima, Kazuhisa Yoshizawa, Masaya Ogasawara, Mizuki Kudo, Yu Itoh, Naoko Ayabe, Nana Shibata, Kazuo Mishima

**Affiliations:** ^1^ Department of Neuropsychiatry, Akita University Graduate School of Medicine, Akita, Japan; ^2^ Department of Regional Studies and Humanities, Faculty of Education and Human Studies, Akita University, Akita, Japan

**Keywords:** antidepressant, antipsychotic, anxiolytic, hypnotic, polypharmacy

## Abstract

**Introduction:**

Guidelines for various psychiatric disorders recommend short-term use of benzodiazepine anxiolytic monotherapy in few cases. Contrarily, benzodiazepine anxiolytic polypharmacy (BAP) is not recommended in any case. However, BAP is often used in real world. Therefore, this study aimed to determine the association between BAP and concomitant use of psychotropic medications.

**Method:**

This retrospective cross-sectional study used claims data from the Japan Medical Data Center. Medical information of health insurance subscribers treated with benzodiazepine anxiolytics in June 2019 was extracted. Prescription of two or more benzodiazepine anxiolytics was defined as BAP. Binary logistic regression analysis was performed to investigate the factors associated with BAP, using age group, sex, type of subscriber, and number of concomitant hypnotics, antidepressants, and antipsychotics (none, one, and two or more) as covariates.

**Result:**

The eligible participants were 104,796 adults who were prescribed benzodiazepine anxiolytics. Among them, 12.6% were prescribed two or more drugs. Logistic regression analysis revealed that BAP was significantly associated with those who received hypnotic monotherapy (adjusted odds ratio [aOR]: 1.04, 95% confidence interval [CI]: 1.001–1.09, p=0.04), antidepressant monotherapy and polypharmacy (aOR: 1.57, 95% CI: 1.51–1.63, p<0.001 and aOR: 1.98, 95% CI: 1.88–2.09, p<0.001, respectively), and antipsychotic monotherapy and polypharmacy (aOR: 1.12, 95% CI: 1.07–1.19, p<0.001 and aOR: 1.41, 95% CI: 1.30–1.54, p<0.001, respectively). Conversely, lower BAP was associated with those who received hypnotic polypharmacy (aOR: 0.86, 95% CI: 0.81–0.91, p<0.001).

**Discussion:**

This study showed that the greater the number of concomitant antidepressants and antipsychotics, the greater the association with BAP. Since combination therapy with antidepressants or antipsychotics is generally not recommended, patients receiving combination therapy with these medications may be resistant to pharmacotherapy. Therefore, implementing the recommended non-pharmacological treatments may reduce BAP.

## 1 Introduction

Benzodiazepines act as positive allosteric modulators of the benzodiazepine-binding sites on GABA_A_ receptors and exert hypnotic, sedative, anxiolytic, and muscle-relaxant effects (1). Benzodiazepine anxiolytics are recommended for short-term monotherapy only in the acute phase of psychiatric diseases or in limited situations according to the guidelines for the treatment of various psychiatric disorders, despite their effectiveness against anxiety symptoms, which are frequent in mental disorders. The Canadian Network for Mood and Anxiety Treatment Guidelines for the Management of Adults with Major Depressive Disorders (MDD) recommend benzodiazepines only for patients with catatonic symptoms (2). The German guidelines for the treatment of anxiety disorders (ADs) allow the use of benzodiazepines only in exceptional cases, such as severe heart disease, suicidality, and contraindications to standard drugs (3). Although the evidence is insufficient (4), the Royal Australian and New Zealand College of Psychiatrists’ clinical practice guidelines for the management of schizophrenia and related disorders suggest the benefit of benzodiazepines for the management of agitation or aggression in patients with schizophrenia and note that they are commonly used (5). Benzodiazepines are not widely recommended in the guidelines for various psychiatric disorders because their efficacy has not been established for schizophrenia or MDD (6, 7). Their short-term efficacy has been shown for panic disorders; however, their long-term efficacy is unknown (8). Long-term use of benzodiazepines can lead to various adverse events, including dependence, falls, fractures, and cognitive impairment (9). However, long-term benzodiazepine prescriptions are common in the real world (10). In addition, elderly patients are at a greater risk of adverse events from benzodiazepines than non-elderly patients because of their increased sensitivity to benzodiazepines and decreased metabolism (11, 12). The American Geriatrics Society Criteria for Potentially Inappropriate Medication Use in Older Adults (American Geriatrics Society Beers Criteria) repeatedly and strongly recommend that older adults avoid benzodiazepine use except in special circumstances such as seizure disorders or alcohol withdrawal (11, 13). However, benzodiazepine prescriptions for the elderly are not uncommon. A national survey of nursing homes in the United States of America (USA) found that 13% of residents used benzodiazepines (14). Benzodiazepine prescription is even more pronounced in older adults exposed to polypharmacy: a cross-sectional study in Norway found that among patients with polypharmacy who were prescribed eight or more medications in primary care, 29.9% were prescribed benzodiazepines or zolpidem (15), and a Slovenian observational study reported that benzodiazepines were the most commonly prescribed potentially inappropriate medication determined with the Potentially Inappropriate Medications in the Elderly list in elderly outpatients with polypharmacy who were prescribed more than 10 medications (16, 17).

Benzodiazepine polypharmacy is an important issue, as is long-term prescription and prescribing to elderly. Benzodiazepine polypharmacy is not uncommon worldwide, and a descriptive cohort study using pharmaceutical claims from a national 10% sample in Australia reported that the prevalence of benzodiazepine polypharmacy was 4.3–2.9% between 2006 and 2015 (18). A nation-wide population-based survey in Taiwan reported that the proportion of person-days of anxiolytic-hypnotic use significantly increased between 2002 and 2009 and that approximately 70% were due to polypharmacy (19). As in other countries, inappropriate use of benzodiazepines is a problem in Japan. A United Nations report in 2010 showed that the average consumption of benzodiazepines in Japan is higher than that in Western and other Asian countries (20). Based on previous reports that medical fee reductions and pharmacist interventions are effective in promoting the appropriate use of benzodiazepines, three policy interventions were implemented in Japan between 2012 and 2018 to correct the inappropriate use of anxiolytics and hypnotics (**Supplementary Table S1**) (21). In the 2012 policy intervention, reimbursement associated with the psychiatry department was reduced for single prescriptions involving three or more anxiolytics or hypnotics. This was further expanded in 2014, when the reimbursement associated with all medical departments was reduced for prescribing three or more anxiolytics or hypnotics in one prescription. In 2018, the reimbursement associated with all departments was reduced to four or more hypnotics and anxiolytics per prescription. In 2018, a new reimbursement was created for patients who received psychotropic polypharmacy when the physician reduced the dose of psychotropic medication and instructed the pharmacist to check for changes in symptoms. However, the effects of these policy interventions were limited, with only a slight decrease in the percentage of patients who were prescribed two or more anxiolytics (11.7% in 2019 compared to 12.7% in 2009) and an increase in the percentage of patients prescribed two or more hypnotics (22.3% in 2019 compared to 19.9% in 2009) (10). To resolve benzodiazepine polypharmacy, the factors associated with benzodiazepine polypharmacy need to be identified and appropriate interventions implemented. Previous studies on benzodiazepine polypharmacy have not distinguished between anxiolytics and hypnotics. Benzodiazepine hypnotics are generally used to treat insomnia, whereas benzodiazepine anxiolytics are used to treat anxiety, agitation, and aggression associated with psychiatric disorders. While some studies have examined the factors associated with psychotropic polypharmacy (22–24) and benzodiazepine sedative-anxiolytics (25), to our knowledge, no study has examined the factors associated with polypharmacy limited to benzodiazepine anxiolytics or benzodiazepine hypnotics. Therefore, an approach that distinguishes between benzodiazepine anxiolytics and hypnotics and examines the factors associated with each type of polypharmacy may be useful.

This study focused on the concomitant use of benzodiazepine anxiolytics (benzodiazepine anxiolytic polypharmacy), which were not recommended by any guideline (2, 3, 5). Identifying factors associated with benzodiazepine anxiolytics polypharmacy may help prevent or correct benzodiazepine anxiolytics polypharmacy. Since benzodiazepine anxiolytics are often prescribed for anxiety, agitation, and aggression associated with psychiatric disorders, we hypothesized that physicians’ prescribing behavior for comorbid psychiatric disorders and the severity of comorbid psychiatric disorders may influence benzodiazepine anxiolytic polypharmacy. In this cross-sectional study, we used claims data to examine the factors associated with benzodiazepine anxiolytic polypharmacy.

## 2 Materials and methods

### 2.1 Study design and dataset

This 1-month retrospective cross-sectional study was a secondary analysis of our previous study (10). In our previous study, we extracted monthly sociodemographic information (age, sex, subscriber type [employees or family members], and prescribed psychotropic medications [benzodiazepine anxiolytics, hypnotics, antidepressants, and antipsychotics]) from the Japan Medical Data Center (JMDC) database (JMDC, Inc, Tokyo, Japan) from April 2005 to June 2019 on January 14, 2020. The JMDC database was created to collect previously unrecorded information to understand the current state of medical care and provide appropriate treatment and information to patients. It is the largest claims database in Japan and contains anonymized claims data from health insurance enrollees since April 2005. The JMDC database generally covers all of Japan; however, the subscribers tend to be in major cities. The subscribers were employees of private companies and their families, aged 0–74 years. The JMDC contains information that hospitals and clinics in Japan can track. On March 21, 2024, this study analyzed the latest June 2019 data from a dataset extracted in our previous study. This study was reported in accordance with the Strengthening the Reporting of Observational Studies in Epidemiology (STROBE) reporting guidelines (26).

### 2.2 Inclusion and exclusion criteria

Patients were eligible if they were 20–74 years old and received a benzodiazepine anxiolytic prescription in June 2019. In Japan, adulthood is defined as an age of ≥20 years until March 31, 2022. Patients aged <20 years and those not prescribed benzodiazepine anxiolytics were excluded. Subscribers not included in the JMDC database in June 2019 were excluded.

### 2.3 Psychotropic drugs

**Table 1** shows the psychotropic medications prescribed under insurance coverage in Japan in June 2019. The JMDC data from June 2019 were used to calculate the number of prescriptions of benzodiazepine anxiolytics, hypnotics, antidepressants, and antipsychotics. Polypharmacy for each psychotropic medication was defined as the prescription of two or more psychotropic medications in June 2019 (18, 27).

**Table 1:** List of psychotropic drugs that can be prescribed in Japan.

*Benzodiazepine anxiolytic*s
Alprazolam, bromazepam, chlordiazepoxide, clorazepate, clotiazepam, cloxazolam, diazepam, etizolam, fludiazepam, flutazolam, flutoprazepam, hydroxyzine, loflazepate, lorazepam, medazepam, mexazolam, oxazepam, oxazolam, prazepam, tofisopam
*Hypnotics*
Amobarbital, barbital, bromovalerylurea, brotizolam, butoctamide, chloral hydrate, estazolam, eszopiclone, etizolam, flunitrazepam, flurazepam, haloxazolam, lormetazepam, nimetazepam, nitrazepam, passiflora extract, pentobarbital calcium, quazepam, ramelteon, rilmazafone, suvorexant, triazolam, zolpidem, zopiclone
*Antidepressants*
Amitriptyline, amoxapine, clomipramine, desipramine, dosulepin, duloxetine, escitalopram, fluvoxamine, imipramine, lofepramine, maprotiline, mianserin, milnacipran, mirtazapine, nortriptyline, paroxetine, safrazine, sertraline, setiptiline, sulpiride, trazodone, trimipramine, venlafaxine
*Antipsychotics*
Aripiprazole, asenapine, blonanserin, blonanserin (tape), brexpiprazole, bromperidol, carpipramine, chlorpromazine, clocapramine, clozapine, floropipamide, fluphenazine, haloperidol, levomepromazine, moperone, mosapramine, nemonapride, olanzapine, oxypertine, paliperidone, perospirone, perphenazine, pimozide, prochlorperazine, propericyazine, quetiapine, risperidone, spiperone, sulpiride, sultopride, tiapride, thioridazine, timiperone, trifluoperazine, zotepine

### 2.4 Statistical analyses

Continuous and categorical variables are expressed as mean and standard deviation (SD) or as numbers and percentages, respectively. The chi-squared test and *post-hoc* comparison z-test with Bonferroni correction were used to compare categorical variables between the monotherapy and polypharmacy groups. A logistic regression model was used to identify factors associated with benzodiazepine anxiolytic polypharmacy (defined as the prescription of two or more benzodiazepine anxiolytics in one month). In the first step, associations between benzodiazepine anxiolytic polypharmacy and explanatory variables (age groups [20–39, 40–64, and 65–74 years], sex, type of subscriber [employees and their family members], and the number of concomitant hypnotics, antidepressants, and antipsychotics [0, 1, 2, or more]) were analyzed separately using univariate logistic regression, and crude odds ratios, 95% confidence intervals (CI), and the related p-values were obtained. In the second step, all variables with a p-value of ≤0.1 in the first step were included in the multivariate logistic models. All statistical analyses were performed using SPSS Statistics version 28.0 (IBMCorp., Armonk, NY, USA). Statistical significance was set at p<0.05 (two-sided).

### 2.5 Ethics

This study was approved by the Ethics Committee of Akita University Graduate School of Medicine (No. 3125, date of approval: March 21, 2024). This study was conducted in accordance with the guidelines of the Declaration of Helsinki. The requirement for informed consent from patients was waived as an anonymized dataset was analyzed in this study.

## 3 Results

**Figure 1:**
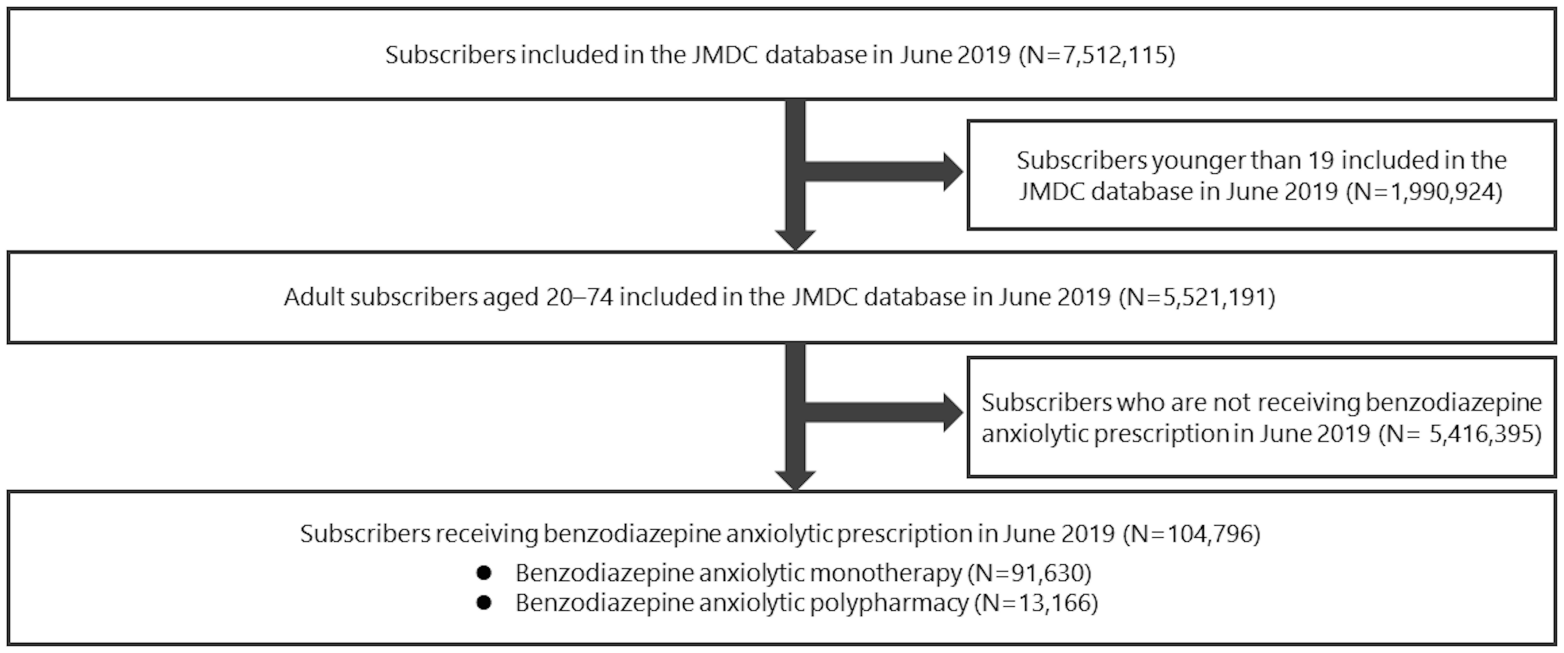
Participant selection flowchart. JMDC, Japan Medical Data Center.

In June 2019, the JMDC database contained the data of 7,512,115 subscribers. Among them, 104,796 adults who were prescribed benzodiazepine anxiolytics were eligible (**Figure 1**). The mean (SD) age was 47.0 ± 11.7 years and 52.8% were female. Among the patients prescribed benzodiazepine anxiolytics, 12.6% were prescribed two or more drugs.

**Table 2** shows the participant characteristics. The percentage of patients aged ≥65 years and who were prescribed benzodiazepine anxiolytics was higher in the monotherapy group (6.3%) than in the polypharmacy group (3.6%). Regarding the concomitant use of other psychotropic medications, the proportion of patients prescribed benzodiazepine anxiolytics and multiple antidepressants was higher in the polypharmacy group than in the monotherapy group (18.5% vs. 12.3%), whereas the proportion of patients prescribed benzodiazepine anxiolytics and multiple antipsychotics was higher in the polypharmacy group than in the monotherapy group (5.5% vs. 3.9%). In contrast, the percentage of patients prescribed benzodiazepine anxiolytics and those taking multiple hypnotics did not differ between the two groups (13.3% in the monotherapy group and 13.5% in the polypharmacy group).

**Table 2:** Demographic and clinical characteristics of participants.

	Total	Benzodiazepine anxiolytic monotherapy	Benzodiazepine anxiolytic polypharmacy	p-value
	N=104,796	N=91,630	N=13,166	
Age, years				<0.001**
20–39	2,7261 (26.0%)	23,751 (25.9%)	3,510 (26.7%)	BAM=BAP
40–64	7,1269 (68.0%)	62,087 (67.8%)	9,182 (69.7%)	BAP>BAM
≥65	6,266 (6.0%)	5,792 (6.3%)	4,74 (3.6%)	BAM>BAP
Sex				<0.001**
Male	49,469 (47.2%)	43,608 (47.6%)	5,861 (44.5%)	
Female	55,327 (52.8%)	48,022 (52.4%)	7,305 (55.5%)	
Subscriber				<0.001**
Him/Herself	67,261 (64.2%)	59,267 (64.7%)	7,994 (60.7%)	
Families	37,535 (35.8%)	32,363 (35.3%)	5,172 (39.3%)	
Hypnotics				<0.001**
0	61,093 (58.3%)	53,770 (58.7%)	7,323 (55.6%)	BAM>BAP
1	29,719 (28.4%)	25,655 (28.0%)	4,064 (30.9%)	BAP>BAM
≥2	13,984 (13.3%)	12,205 (13.3%)	1,779 (13.5%)	BAM=BAP
Antidepressants				<0.001**
0	55,015 (52.5%)	49,576 (54.1%)	5,439 (41.3%)	BAM>BAP
1	36,064 (34.4%)	30,767 (33.6%)	5,297 (40.2%)	BAP>BAM
≥2	13,717 (13.1%)	11,287 (12.3%)	2,430 (18.5%)	BAP>BAM
Antipsychotics				<0.001**
0	87,739 (83.7%)	77,172 (84.2%)	10,567 (80.3%)	BAM>BAP
1	12,768 (12.2%)	10,890 (11.9%)	1,878 (14.3%)	BAP>BAM
≥2	4,289 (4.1%)	3,568 (3.9%)	721 (5.5%)	BAP>BAM

Values are presented as numbers (%); p-values with significant results (<0.001) are labeled with a double asterisk.

BAM, benzodiazepine anxiolytic monotherapy; BAP, benzodiazepine anxiolytic polypharmacy.

**Table 3** shows the results of the logistic regression analysis. In the univariate analysis, all factors were associated with benzodiazepine anxiolytic polypharmacy. Polypharmacy of benzodiazepine anxiolytics was significantly associated with middle age (compared with younger age) (adjusted odds ratio [aOR]: 1.05, 95% CI: 1.01–1.09, p=0.03) and female sex (aOR: 1.10, 95% CI: 1.05–1.15, p<0.001). Significant associations were also observed in families than in subscribers (aOR: 1.18, 95% CI: 1.12–1.23, p<0.001) and those who underwent hypnotic monotherapy (aOR: 1.04, 95% CI: 1.001–1.09, p=0.04), antidepressant monotherapy and polypharmacy (aOR: 1.57, 95% CI: 1.51–1.63, p<0.001 and aOR: 1.98, 95% CI: 1.88–2.09, p<0.001, respectively), and antipsychotic monotherapy and polypharmacy (aOR: 1.12, 95% CI: 1.07–1.19, p<0.001 and aOR: 1.41, 95% CI: 1.30–1.54, p<0.001, respectively). Conversely, lower polypharmacy of benzodiazepine anxiolytics was associated with older adults and those who were prescribed two or more hypnotics (aOR 0.63, 95% CI: 0.57–0.70, p<0.001 and aOR: 0.86, 95% CI: 0.81–0.91, p<0.001, respectively).

**Table 3:** Factors associated with anxiolytics polypharmacy.

	Crude OR (95%CI)	p-value	Adjusted OR (95%CI) ^†^	p-value
Age, years				
20–39	Reference		Reference	
40–64	1.00 (0.96–1.04)	0.97	1.05 (1.01–1.09)	0.03*
≥65	0.55 (0.50–0.61)	<0.001**	0.63 (0.57–0.70)	<0.001**
Sex				
Male	Reference		Reference	
Female	1.13 (1.09–1.17)	<0.001**	1.10 (1.05–1.15)	<0.001**
Subscriber				
Him/Herself	Reference		Reference	
Families	1.19 (1.14–1.23)	<0.001**	1.18 (1.12–1.23)	<0.001**
Hypnotics				
0	Reference		Reference	
1	1.16 (1.12–1.21)	<0.001**	1.04 (1.001–1.09)	0.04*
≥2	1.07 (1.01–1.13)	0.02*	0.86 (0.81–0.91)	<0.001**
Antidepressants				
0	Reference		Reference	
1	1.57 (1.51–1.63)	<0.001**	1.57 (1.51–1.63)	<0.001**
≥2	1.96 (1.86–2.07)	<0.001**	1.98 (1.88–2.09)	<0.001**
Antipsychotics				
0	Reference		Reference	
1	1.26 (1.19–1.33)	<0.001**	1.12 (1.07–1.19)	<0.001**
≥2	1.48 (1.36–1.60)	<0.001**	1.41 (1.30–1.54)	<0.001**

p-values with significant results (<0.05) are labeled with an asterisk, and those with significant results (<0.001) are labeled with a double asterisk. The larger the odds ratio, the stronger the positive association with benzodiazepine anxiolytic polypharmacy.

^†^Adjusted for age groups (20–39, 40–64, and 65–74 years), sex, type of subscriber (employees and their family members), and the number of concomitant hypnotics, antidepressants, and antipsychotics (0, 1, 2 or more).

CI, confidence interval; OR, odds ratio.

## 4 Discussion

This study examined the factors associated with polypharmacy of benzodiazepine anxiolytics. The primary finding of this study was that benzodiazepine anxiolytic polypharmacy was positively associated with concomitant use of antidepressants and antipsychotics, consistent with a previous study of elderly people in Sweden that reported an association of polypharmacy with benzodiazepines, benzodiazepine-related drugs (including hypnotics and anxiolytics), and concomitant antipsychotic and antidepressant medication (25). Notably, this study found that the association with benzodiazepine anxiolytic polypharmacy increased as the number of concomitant antidepressants and antipsychotics increased.

Among psychotropic medications, antidepressants are most strongly associated with benzodiazepine anxiolytic polypharmacy. Benzodiazepine anxiolytic polypharmacy was approximately 1.6 times higher in patients who received antidepressant monotherapy and approximately twice as high in those who received antidepressant polypharmacy than in those who did not. Antidepressants are the first-line treatment for AD and MDD (2, 3), and anxiety may be the primary symptom. Most guidelines recommend antidepressant monotherapy for these disorders. However, antidepressant monotherapy is sometimes insufficient to treat these mental disorders. For MDD, combination antidepressant therapy may be used as an adjunctive therapy when antidepressant monotherapy is not effective (18, 28, 29). In fact, the concomitant use of some combinations of antidepressants is recommended only when first-line treatment fails to produce adequate improvement (2, 3). Therefore, patients receiving antidepressant polypharmacy may have included more patients who did not respond adequately to antidepressants than those who were prescribed antidepressant monotherapy. Physicians may have prescribed multiple benzodiazepines to patients with antidepressant resistance knowing that there was no evidence of efficacy or a high risk of side effects. This study did not extract information from the JMDC database on electroconvulsive therapy, which is often used to treat refractory MDD. A multicenter study in Japan reported that patients hospitalized for MDD and treated with electroconvulsive therapy had lower rates of anxiolytic medication use at discharge than those who were not treated (30). Therefore, patients with MDD who have had an inadequate response to guideline-directed pharmacotherapy should not continue treatment with pharmacotherapy alone; however, electroconvulsive therapy may be considered as a treatment option. This may help patients to avoid psychotropic polypharmacy. Cognitive behavioral therapy (CBT) is another factor that may have influenced the results of this study and could not be controlled. CBT is a typical psychotherapy with established efficacy and safety for both AD and MDD (31–33);, and it may improve psychiatric symptoms in patients whose AD and MDD do not improve with antidepressant monotherapy (34). Therefore, implementing CBT for depression and anxiety may prevent prescription of benzodiazepine anxiolytics and their polypharmacy. In addition, because CBT can assist in benzodiazepine discontinuation (35), CBT for AD and MDD may discourage the polypharmacy of benzodiazepine anxiolytics. Therefore, it is important to consider evidence-based non-pharmacological therapies for the treatment of mental disorders.

Notably, compared with patients who were not prescribed hypnotics, those who were prescribed one hypnotic showed a positive association with benzodiazepine anxiolytic polypharmacy, whereas those who were prescribed hypnotic polypharmacy showed a negative association. The higher the number of concomitant antidepressants and antipsychotics, the higher the risk of benzodiazepine anxiolytic polypharmacy, but not that of hypnotics. This may have been influenced by Japan’s policy interventions in April 2018. Prior to April 2018, medical fees were not reduced for prescribing two anxiolytics and two hypnotics in a prescription; after April 2018, medical fees were reduced for prescribing four or more hypnotics and anxiolytics in a single prescription (10). This study suggests that policy interventions may have led to a change in physician-prescribing behaviors, such as their attempts to avoid hypnotic polypharmacy in patients undergoing benzodiazepine anxiolytic polypharmacy to avoid a reduction in reimbursement. To date, no longitudinal study has been conducted on the impact of the April 2018 policy intervention on prescribing more than four prescriptions of anxiolytics and hypnotics; therefore, further well-designed research is needed to clarify the effects of the 2018 policy intervention.

The patient characteristics associated with benzodiazepine anxiolytic polypharmacy included older age, female sex, and being a family member of a subscriber. It is understandable that older patients are less likely to be prescribed benzodiazepine anxiolytic polypharmacy, as they are generally more susceptible to side effects than younger patients (36). No studies have examined the association between sex or occupation and benzodiazepine anxiolytic polypharmacy, and the results of previous studies examining the factors associated with psychotropic medication polypharmacy are inconsistent (22, 27, 37). Since this study lacks information on marital status, cohabitants, and income, which have been examined in previous studies, future studies with sophisticated designs are needed to clarify the association between benzodiazepine anxiolytic polypharmacy and sociodemographic factors.

Many studies have examined psychotropic polypharmacy using various definitions (e.g., concomitant use of psychotropic medications of the same or different classes) in various populations (e.g., the elderly, children, adolescents, patients with bipolar disorders, and patients with mental retardation) (22–25, 38). Previous studies have shown that psychotropic polypharmacy is associated not only with age, sex, and concomitant psychotropic medications but also with numerous other factors not considered in this study, including marital status, comorbid psychiatric disorders, and the number of non-psychotropic medications prescribed (22–25). A Swedish study of adults aged 75 years and older found that the rate of concomitant prescription of two or more benzodiazepines or benzodiazepine-related medications (including hypnotics and anxiolytics) was 4.7%, with older age, female sex, urban setting, number of prescribed non-psychiatric medications, antidepressant use, and antipsychotic use being associated with polypharmacy (25). A study of patients attending outpatient psychiatric clinics in Saudi Arabia that defined psychotropic polypharmacy as the prescription of two or more psychotropic medications reported a prevalence of psychotropic polypharmacy of 46.9%, with older age, divorce, and diagnoses of psychosis and bipolar disorder associated with psychotropic polypharmacy (22). A study on bipolar disorder in Europe that defined psychotropic polypharmacy as the prescription of four or more psychotropic medications reported that the prevalence of psychotropic polypharmacy was 40.5%, and single status, older age, number of hospitalizations, and presence of psychiatric comorbidities were associated with polypharmacy (24). Therefore, future studies are needed to investigate factors associated with benzodiazepine anxiolytic polypharmacy, taking these factors into account.

This 1-month retrospective cross-sectional study found that 12.6% of adults prescribed benzodiazepine anxiolytics received benzodiazepine anxiolytic polypharmacy. A previous study of older people in Sweden reported that 19.1% of patients prescribed benzodiazepines or benzodiazepine-related drugs had polypharmacy with two or more of these drugs (25). There were differences in patients’ mean ages (47.0 years in this study and 82.1 years in the study conducted in Sweden) and definition of benzodiazepine polypharmacy (polypharmacy with benzodiazepine anxiolytics vs. polypharmacy with benzodiazepines and benzodiazepine-related drugs) between both studies. In both studies, benzodiazepine polypharmacy was common. Interestingly, a nationwide, population-based survey in Taiwan aimed at assessing anxiolytic-sedative use using person-days (which takes into account prescription duration not examined in this study) showed greater rates of hypnotic anxiolytic-sedative effects in patients prescribed hypnotic sedatives (19). Therefore, future studies are needed to examine factors associated with benzodiazepine anxiolytic polypharmacy while considering the duration of benzodiazepine anxiolytic prescriptions.

Pharmacist intervention is a potential solution for benzodiazepine anxiolytic polypharmacy (39–41). Pharmacist intervention is effective not only against benzodiazepine deprescribing (42), but also against polypharmacy of antipsychotics and antidepressants (43–45). Since concomitant psychotropic medications were associated with polypharmacy with benzodiazepine anxiolytics in this study, pharmacist intervention may not only work directly against benzodiazepine anxiolytic polypharmacy, but also indirectly by appropriately guiding psychotropic medications other than benzodiazepine anxiolytics. In Japan, a new reimbursement system was created in April 2018 for pharmacists’ cooperation in reducing psychotropic polypharmacy at the request of physicians. To date, the extent to which physician–pharmacist collaboration on the reduction of psychotropic polypharmacy based on this 2018 policy intervention has occurred and how effective it has not been studied. However, further studies are warranted to confirm these findings.

This study had some limitations. First, the extent to which the JMDC data set represents the general Japanese population remains unclear. Since the database is based primarily on health insurance associations belonging to large corporations, its members are more likely to be in large cities and less likely to be in regional cities. In addition, the JMDC database does not include data on subscribers aged ≥75 years. Therefore, the data used may have been skewed due to sociodemographic and geographical factors. Second, because this was a cross-sectional study, the causal relationship between anxiolytic polypharmacy and related factors remains unknown. Therefore, prospective cohort studies are required to clarify these causal relationships. Third, the data used were based only on a limited timeframe, and the results may have been different if other periods were used. Fourth, the number of psychotropic medications prescribed per month was calculated. Therefore, the number of drugs administered may have been overestimated, even if the number of drugs administered after switching to psychotropics in the same category in the same month remained the same. Thus, this study may have overestimated polypharmacy rates. Fifth, this study lacked data on the diagnosis and severity of mental disorders and information on who was prescribed psychotropics (psychiatrists or non-psychiatrists). Sixth, because this study did not extract diagnoses of psychiatric or physical illness, we could not examine the clinical indications for psychotropic medications, including benzodiazepine anxiolytics. Seventh, the results are specific to Japan and may not apply to other parts of the world. Eighth, apart from the 2018 policy intervention, there may be several reasons behind the results of this study that could not be considered and may have influenced the results of this study. Ninth, this study lacked data on drug–drug interactions, drug metabolism, and excretion. In addition, this study examined whether individual patients were prescribed psychotropic medications and the number of medications prescribed, but not their blood concentrations. These factors may have affected the results of this study.

In conclusion, this study suggests that patients prescribed two or more antidepressants or antipsychotics are more likely to be at risk for benzodiazepine anxiolytic polypharmacy. Since combination therapy with antidepressants or antipsychotics is generally not recommended by the guidelines, patients receiving combination therapy with these medications may be resistant to pharmacotherapy (2, 3, 5). Therefore, implementing the recommended non-pharmacological treatments for each psychiatric disorder may reduce benzodiazepine anxiolytic polypharmacy. In addition, the negative association between benzodiazepine anxiolytics and hypnotic polypharmacy may be influenced by policy interventions for four or more anxiolytics and hypnotics. Future studies should examine the risk of benzodiazepine anxiolytic polypharmacy, considering the reasons for the concomitant use of benzodiazepine anxiolytics, severity of psychiatric disorders, history of non-pharmacological treatment, physician prescription patterns, and patient treatment preferences.

## Data availability statement

Data supporting the findings of this study are available from the JMDC. However, restrictions apply to the availability of these data. The data were used under license for the current study, and thus are not publicly available. The raw data supporting the conclusions of this article will be made available by the authors, without undue reservation, and with permission from JMDC.

## Ethics statement

The studies involving humans were approved by The Ethics Committee of Akita University Graduate School of Medicine. The studies were conducted in accordance with the local legislation and institutional requirements. The ethics committee/institutional review board waived the requirement of written informed consent for participation from the participants or the participants’ legal guardians/next of kin because The requirement for informed consent from patients was waived as an anonymized dataset was analyzed in this study.

## Author contributions

MT: Writing – original draft, Visualization, Validation, Software, Resources, Project administration, Methodology, Investigation, Formal analysis, Data curation, Conceptualization. KY: Writing – original draft, Methodology, Formal analysis, Data curation, Conceptualization. MO: Writing – original draft, Methodology, Investigation, Formal analysis, Data curation, Conceptualization. MK: Writing – original draft, Methodology, Formal analysis, Data curation, Conceptualization. YI: Writing – original draft, Methodology, Formal analysis, Data curation, Conceptualization. NA: Writing – original draft, Methodology, Formal analysis, Data curation, Conceptualization. NS: Writing – original draft, Methodology, Formal analysis, Data curation, Conceptualization. KM: Writing – original draft, Supervision, Methodology, Formal analysis, Data curation, Conceptualization.
