# Supplementary material for: Association between benzodiazepine anxiolytic polypharmacy and concomitant psychotropic medications in Japan: a retrospective cross-sectional study
Source: Front Psychiatry. 2024 Jul 4;15:1405049. doi: 10.3389/fpsyt.2024.1405049 (PMC11254787; doi:10.3389/fpsyt.2024.1405049)
Supplement: Supplementary file 1 [file Table_1.pdf]

Table S1. Details of the medical fee revisions aimed at reducing anxiolytic and hypnotic polypharmacy in Japan

Year of revision	Requirements	Extent of the revision
2012	Administration of three or more anxiolytics or three or more hypnotics in one prescription	Subsidy for continuous psychiatric outpatient services/ consultation fee (55 points/day)<br>➡ Calculated as 80 out of 100 points
2014	Administration of three or more anxiolytics, three or more hypnotics in one prescription	Subsidy for transportation costs and consultation fee for psychiatric treatments<br>➡ Incalculable<br>Outpatient prescription charges: 68 points ➡ 30 points<br>Inpatient prescription charges: 42 points ➡ 20 points<br>Medical fees: Calculated as 80 out of 100 points
2016	Administration of three or more anxiolytics, three or more hypnotics in one prescription	Inpatient and outpatient prescription charges and medical fees did not change
2018	Administration of three or more anxiolytics, three or more hypnotics, or four or more anxiolytics and hypnotics in one prescription	Outpatient prescription charges: 68 points ➡ 28 points<br>Inpatient prescription charges: 42 points ➡ 18 points<br>Medical fees: Calculated as 80 out of 100 points
	When the physician reduces the dose of the psychotropic medication and instructs the pharmacist to check for changes in symptoms	Outpatient prescription charges: 68 points ➡ 80 points<br>Inpatient prescription charges: 42 points ➡ 54 points

Note: One point=10 Japanese yen
